# Opportunities and Challenges for Precision Nutrition in Gestational Obesity Management

**DOI:** 10.1007/s13679-026-00710-x

**Published:** 2026-04-02

**Authors:** Emmie Söderström Shields, Nina Kaegi-Braun, Johanna Sandborg, Caroline Lilliecreutz, Marie Löf

**Affiliations:** 1 Department of Medicine, Huddinge, Karolinska Institutet, Stockholm, 141 86 Sweden; 2 Department of Obstetrics and Gynecology, Linköping University Hospital, and Department of Biomedical and Clinical Sciences, Linköping University, Linköping, Sweden

**Keywords:** Precision nutrition, Risk stratification, Risk profiling, Obesity management, Maternal obesity, Gestational weight gain, Lifestyle interventions, Metabolic status

## Abstract

**Purpose of Review:**

This review summarizes current knowledge on precision nutrition in gestational obesity management and outlines priorities for future research and implementation.

**Recent Findings:**

We summarize evidence on key factors (i.e., observable traits, social determinants of health, behavioral factors, clinical biomarkers, tissue-specific features, molecular-level data) that can be assessed and targeted within a precision nutrition approach for managing gestational obesity. Recent studies highlight obesity classes, behavioral-metabolic obesity phenotyping (hungry brain, emotional hunger, hungry gut) and the maternal metabolic milieu as interesting targets. Women’s and healthcare professionals’ (end-users) needs and perceptions as well as digital infrastructure requirements need further exploration.

**Summary:**

There is promise for more precision-based nutrition in optimizing gestational obesity management moving away from current one-size-fits all approaches. However, additional research is required to determine appropriate precision‑based nutrition approaches, evaluate their efficacy and cost‑effectiveness, and develop strategies for their implementation in routine care.

## Introduction

Obesity remains a major public health challenge worldwide, and to date no country has successfully curbed the rising prevalence of adult obesity [1]. The situation also applies to pregnant women with 16% globally entering gestation with obesity [2]. This is a matter of serious concern considering that pre-pregnancy obesity is associated with increased risks of many adverse maternal and neonatal outcomes including gestational diabetes mellitus (GDM), pre-eclampsia, large-for-gestational age (LGA) infants, and breastfeeding initiation challenges [3]. Long-term consequences encompass higher maternal cardiometabolic risk and increased risk of childhood obesity [4, 5].

Management of obesity during pregnancy (i.e., gestational obesity) in maternity healthcare typically includes interventions to improve diet and physical activity and to prevent excessive gestational weight gain (GWG) through individual or group counselling. Lifestyle interventions have been shown to reduce excessive GWG and improve diet quality during pregnancy [6], also in women with obesity [7]. However, effect sizes have been relatively small [6, 7], and most programs follow a “one-size-fits-all” approach. Furthermore, these interventions are resource-intensive and challenging to scale up, motivating further research on more effective interventions that can be integrated into routine maternity healthcare.

Addressing these challenges requires innovative strategies moving beyond standardized interventions and accounting for individual differences in physiology, lifestyle, and context. One emerging concept that aligns with this need is *precision nutrition*, which offers a framework for risk stratification and tailoring dietary interventions based on multidimensional data. While the term has been used inconsistently, often referring broadly to personalized dietary advice, Da Silva et al. [8] propose a conceptual framework integrating multidimensional data such as phenotype, behavioral, clinical, tissue-specific and molecular information to enable biologically meaningful stratification or guide individualized dietary recommendations. It builds on the understanding that variations in clinical characteristics, psychosocial factors, diet, microbiota, and genetics influence nutritional responses. Emerging evidence suggests the potential of precision nutrition also in pregnancy, e.g., based on observed heterogeneity in the association between dietary patterns and adverse outcomes [9]. This review summarizes current evidence on precision nutrition in gestational obesity management and outlines priorities for future research and implementation.

### Pathophysiology: Links between Gestational Obesity and Adverse Outcomes

Understanding the biological mechanisms linking obesity to adverse maternal and fetal outcomes is critical for identifying intervention targets and provides a foundation for precision nutrition strategies to modulate these pathways. These mechanisms are complex and multifactorial, involving three interconnected compartments, mother, fetus and the placenta, and vary across the preconception period and throughout pregnancy. Multiple organ systems and biological pathways contribute to the underlying pathophysiology, including metabolic [10, 11], endocrine [12], inflammatory [13, 14] and vascular [15] processes. The placenta has a key role by facilitating maternal-fetal nutrient transfer, through hormones that increase insulin resistance and secretion [16]. In women with obesity, pre-existing hyperinsulinism increases nutrient availability, contributing to excessive fetal growth and related short-term maternal and neonatal complications [17]. Additional obesity-associated conditions such as inflammation, dyslipidemia, impaired endothelial function, hypertension, and oxidative stress may also impair placental function [11, 15, 17] although the precise biological mechanisms remain unclear.

### Potential of Precision Nutrition in Gestational Obesity Management

The goal of precision nutrition is to deliver more accurate and scalable dietary interventions by combining mechanistic insights with practical applicability. Da Silva et al. [8] presents a structured framework of data required to implement precision nutrition strategies, organized into different levels with each reflecting increasing biological granularity.

Specifically:

- **Macro-level data** encompasses observable traits such as age and BMI.
- **Between macro and micro level data** comprises data on behavior and lifestyle.
- **Micro-level data** includes clinical biomarkers and tissue-specific characteristics.
- **Molecular-level data** pertains to omics information.

**Fig. 1:**
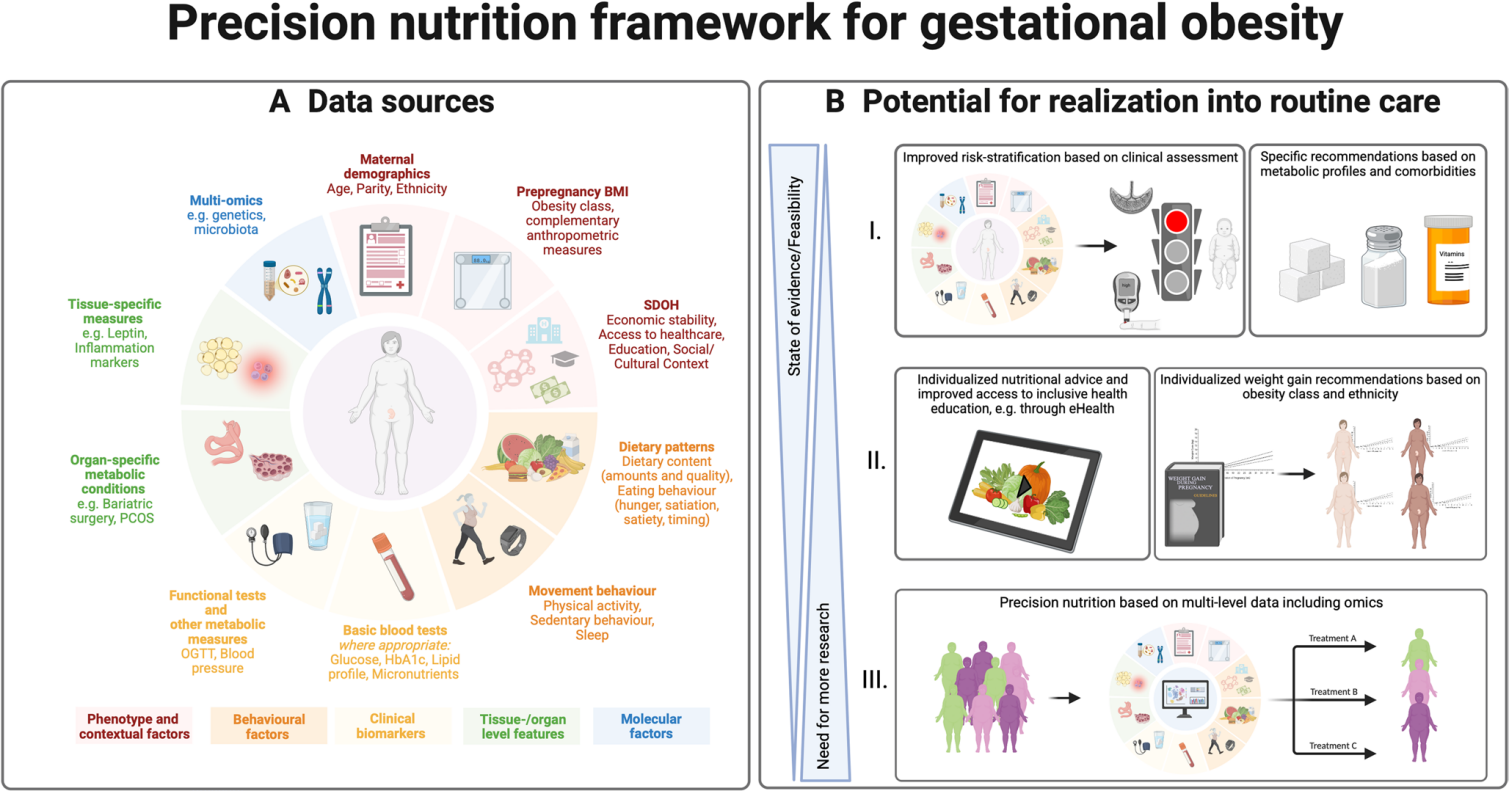
(**A**) Proposed data sources to assess in order to apply a potential precision nutrition approach for managing obesity during pregnancy based on the framework and data level categorization published by Da Silva et al. [18]. (**B**) Potential for realization into routine care, with progression illustrated across levels I–III according to feasibility and state of evidence

Building on this framework, we outline key factors to assess for applying a potential precision nutrition approach in managing obesity during pregnancy (Fig. 1A). Table 1 summarizes examples from the literature supporting risk stratification and individualized dietary advice, and outlines current implications for precision nutrition. Below, we comment on each data level, discussing potential, supporting evidence, and research gaps.

**Table 1:** Examples of data to be collected for a precision nutrition approach for gestational obesity management

Level of data to collect	Description	Assessment method	Clinical practice routines in maternity healthcare	Examples from the literature		Current implications for precision nutrition in maternity healthcare*
				Stratification into meaningful sub-groups (Identification of high-risk populations)	Guidance of individualized dietary advice	
**Maternal observable traits and social determinants of health**						
Maternal age and parity	Age at conception and number of previous live births	Clinical records	Routinely assessed	Combined exposure to advanced maternal age and pre-pregnancy overweight/obesity was associated with a higher risk of pre-eclampsia and GDM than either factor alone, e.g. for preeclampsia, the odds ratio was 3.82 when comparing normal‑weight women < 35 years with overweight/obese women < 35 years, and 5.66 when comparing normal‑weight women < 35 years with overweight/obese women > 35 years [18]. Positive association between parity and early-pregnancy insulin resistance aggravated in women with gestational overweight/obesity [20]. No association was found between multiparity and type 2 diabetes risk in normal‑weight women, whereas in women with obesity, having four or more children was associated with a higher type 2 diabetes risk (HR 1.41) [21]	-	Feasible for individualized risk assessment; implications for dietary management remain unclear
Pre-pregnancy BMI	Obesity classes I-III	Self-reported/ measured (weight/height)	Routinely calculated (BMI)	Pre-pregnancy obesity class is associated with a series of adverse maternal and fetal outcomes, e.g. LGA rates were 49% more likely in women with class III compared to women with class I obesity [25, 26]	BMI currently serves as foundation for individualized weight gain guidelines, but no standardized guidelines on how to tailor dietary advice (e.g., energy intake) to pre-pregnancy BMI [24]	Feasible for individualized risk assessment; possible implication of obesity classes for future GWG guidelines and dietary strategies. Future obesity assessment might include other anthropometric measures along BMI
Ethnicity and SDOH	Subgroup that shares common descent or cultural background, economic stability (food insecurity); education access and quality; healthcare access and quality; neighborhood and built environment; social and community context	Self-reported, validated questionnaires	Assessment frequency and level of detail unknown and likely considerably variable across settings	Ethnic variations in population-level effects of pre-pregnancy obesity and excessive GWG on the risk of LGA [31]. Obesity prevalence is higher in food‑insecure pregnant women than in food‑secure women (40.8% vs. 24.7%), and food insecurity is associated with increased perinatal complication risk even after adjusting for BMI [36]	Ethnicity-specific BMI categories to define weight gain targets in women from Asia [32]. Effect modification of food assistance programs on the association between food insecurity and perinatal complications [36]	Feasible for individualized risk assessment; shift towards ethnicity-specific categorization for GWG recommendations. Evidence for SDOH-, and ethnicity-specific dietary interventions remains limited. Research about tailored nutrition interventions (including digital format) is ongoing but implementation remains context- and resource-dependent
**Behavioral factors**						
Dietary patterns	Intake of foods, macro/micronutrients	Questionnaires, interviews, digital assessment tools	Diet routinely assessed but only brief screening questions	Poor or inadequate maternal nutrition is linked to abnormal fetal growth patterns, which in turn are associated with an increased risk of chronic diseases in childhood and adulthood	Certain dietary patterns during pregnancy are associated with a lower risk of excessive GWG (limited evidence). These patterns are higher in vegetables, fruits, nuts, legumes, fish, and lower in added sugar, and red and processed meat [84]	Feasible for individualized risk assessment; detailed assessment limited by time constraints. Baseline nutrient intake can guide targeted dietary adjustments, although validated nutrient-specific precision strategies in pregnancy are limited
	Eating behavior	Questionnaires assessing eating behaviors (hunger, satiation and satiety)	Not assessed in routine care	An obesity phenotype guided program (based on pathophysiology impaired satiation, hedonic eating, impaired satiety) resulted in higher weight loss [39]. ** Chrononurition: maternal nighttime eating associated with adverse pregnancy outcomes, such as higher preterm birth risk, impaired glucose metabolism, poor sleep quality, higher GWG, and higher risk of postpartum weight retention [40]	A chrononutritional and sleep hygiene intervention can improve maternal glycemic control in GDM patients [41]	Feasible for individualized risk assessment; Emerging concepts for potential phenotype-based dietary strategies, although limited evidence
Movement behaviors	Level of physical activity, sedentary behavior and sleep	Questionnaire, accelerometers	not routinely assessed	Achieving 10 MET‑h/week in early pregnancy reduces GDM risk by 13%, and higher activity volumes (20–50 MET‑h/week) confer further reductions [44]. In a cohort of pregnant women, sleep duration showed a U‑shaped association with glycemia, and greater sedentary time was associated with higher 1‑hour post‑load glucose (β = 0.132; *P* = 0.005) [45]	-	Feasibility limited by time and resources for movement behavior assessment; no individualized dietary strategies yet.
**Clinical biomarkers**						
Metabolic status (specific biomarkers are described below)	Metabolically healthy/unhealthy obesity	Blood tests (fasting glucose, HbA1c, lipid profile, HOMA-IR)	Not measured but some of the variables are routinely assessed	Metabolic milieu is a better risk marker for adverse pregnancy outcomes than GWG. For instance, higher birth weight and fat mass observed in offspring [46]	Targeting glucose, triglyceride, and leptin levels or inflammatory makers improves the metabolic milieu and overall health [46]**	Pathophysiological insights and hypothesis-generation; metabolic phenotyping has potential for risk stratification but evidence linking specific phenotypes to differential dietary responses in pregnancy are limited
Glucose parameters	Pre-existing glucose intolerance or GDM	Blood test (HbA1c, Fasting glucose) OGTT Finger-prick capillary glucose self-measurements	Different screening and diagnosis regimes. ADA guidelines [50]: Test individuals planning pregnancy or pregnant women < 15 weeks with overweight or obesity plus an additional risk factor (consider testing all individuals) for undiagnosed pre-diabetes and diabetes using standard diagnostic criteria (fasting glucose, HbA1c). Screen for GDM at 24–28 weeks of gestation	Pre-existing diabetes is associated with significant maternal and neonatal risk, e.g. OR for preeclampsia and cesarean delivery 3.5, OR for cardiac congenital defects 4.6, OR for stillbirth 3.5, OR for perinatal mortality 3.4 [51]. Offspring of women with pre‑existing type 2 diabetes have the highest risk of perinatal mortality compared with other diabetes types; compared with offspring of non‑diabetic women, the odds ratios are 4.2 for perinatal mortality and 7.3 for stillbirth [52]. Overweight is one of the strongest risk factors for GDM (OR 5.6), GDM is associated with increased risk of caesarean section (OR 1.5), preterm delivery (OR 1.6), postpartum depression (OR 4.6), and breastfeeding complications [53]	In women with GDM, modified dietary interventions (pooled analysis of low glycemic index, DASH, low carbohydrate, fat modification, soy protein-enrichment, behavior intervention, ethnic diets) favorably influenced maternal glucose outcomes and birth weight (mean difference − 170 g and relative risk for macrosomia 0.5) [55]	Feasible and important biomarker for individual risk stratification; regional differences in screening regimes and diagnostic approaches. Dietary advice and insulin treatment are individualized based on glucose monitoring patterns, whereas structured precision nutrition strategies and macronutrient recommendations remain insufficiently investigated.
Blood pressure	Pregnancy induced hypertension, essential hypertension, preeclampsia, eclampsia, HELLP syndrome	Sphygmomano-meter	Routinely assessed	Hypertensive disorders during pregnancy are associated with adverse maternal and perinatal outcomes [60]	Higher DASH dietary scores were linked to lower mid-pregnancy diastolic BP and better fetoplacental vascular function [61]	Feasible for individual risk stratification; an established tailored dietary intervention (DASH) is recommended in the non‑pregnant population, with growing evidence supporting its effectiveness in pregnant women as well.
Lipid profile	Dyslipidemia during pregnancy	Blood test (total cholesterol, LDL, HDL, triglycerides)	Not commonly assessed in routine care. No general lipid screening recommendation during pregnancy [59]	Increased risk for adverse pregnancy outcomes, as well as pancreatitis in the presence of severe hypertriglyceridemia [57, 58]	Same dietary interventions as for non-pregnant individuals with dyslipidemia but avoiding too strict diets [59]	Feasible for individual risk stratification and tailored interventions but not currently universally implemented.
**Tissue-/organ specific level features**						
PCOS	PCOS subtype based on BMI, fasting glucose, insulin, reproductive hormones and androgens	Clinical records	Subtypes not assessed in routine care	PCOS and certain PCOS subtypes may increase risk of pregnancy complications. One subtype characterized by obesity and metabolic dysfunction (OB-PCOS), is associated with adverse outcomes including increased risk of GDM and hypertension [62]	-	Use for risk stratification still in research stage; Potential additional information relevant for recognizing high-risk subgroups but no precision nutrition strategies available
Bariatric surgery	Anatomical changes resulting in malabsorption. Possible nutritional deficiencies and non-beneficial eating behaviors in women with prior bariatric surgery	Clinical records	Clinical and biochemical assessments recommended before and during pregnancy	Women with prior bariatric surgery and those with GDM represent relevant subgroups requiring tailored nutritional strategies [65, 66]	Personalized nutritional counseling can improve diet quality and birth weight [66]. Individualized nutritional guidelines for energy requirement and macronutrient guidance [65]. Specific recommendations regarding micronutrient screening (preconceptionally and at least once per trimester) and supplementation (e.g. folic acid) [67]	Feasible for individual risk stratification; Precision-aligned strategies for handling the condition already implemented with established individualized nutritional monitoring and supplementation protocols
**Molecular-level data**						
Genetic predisposition and nutrigenetics	Genetic variations, diet-gene interaction, epigenetics.	Genetic analysis, polygenic risk scores, GWAS panels	Not assessed in routine care	Genetic predisposition to central fat distribution associated with increased risk of pre-eclampsia [68]. Carrying multiple risk alleles may predict susceptibility to GDM [70]. Associations between C677T (polymorphism in the gene encoding the folate-metabolizing enzyme methylene-etetrahydrofolate reductase) and increased risk of hypertensive disorders of pregnancy [69]	Carriers of APOA1 variants show improved HDL cholesterol after PUFA-rich diets, and APOE ε4 carriers benefit from reduced saturated fat, while variants in FADS genes affect omega-3 metabolism, suggesting tailored supplementation could optimize lipid and inflammatory profiles [75].** Supplemental riboflavin can effectively lower blood pressure specifically in individuals with the variant *MTHFR* 677TT genotype, but the role for hypertensive disorders during pregnancy need to be evaluated [72]**	Implication for risk stratification have been shown in research but not clinically feasible at the moment; gene-based dietary personalization in pregnancy remains insufficiently studied
Gut microbiota	Dysbiosis during pregnancy, altered SCFA-producing bacteria. May influence energy harvest, insulin sensitivity and inflammation	Stool samples, sequencing	Not assessed in routine care	Changes in microbial diversity and abundance, and the presence of intestinal dysbiosis, are associated with higher obesity severity and increased GDM risk [77]	High fiber diet enhances microbiota diversity and high-fat diets promote inflammation [79]. Probiotic supplementation and implementing fiber interventions may improve glycemic control [78]	Implication for risk stratification have been shown in research but not clinically feasible at the moment; no clinically implementable microbiota-based dietary strategies in pregnancy

The list of precision nutrition targets in this table is not exhaustive, and the references provided are intended as illustrative examples rather than a comprehensive compilation

ADA, American Diabetes Association; BMI, body mass index; BP, blood pressure; CRP, C-reactive protein; DASH (Dietary Approaches to Stop Hypertension); GDM, gestational diabetes mellitus; GWAS, genome-wide association studies; GWG, gestational weight gain; HDL, high-density lipoproteins; HELLP, Hemolysis, Elevated Liver enzyme levels, and Low Platelet levels; HOMA-IR, homeostasis model assessment-estimated insulin resistance; HbA1c, hemoglobin A1C; IL-6, interleukin 6; LDL, low-density lipoproteins; LGA, large for gestational age; PCOS, Polycystic Ovary Syndrome; OGTT, oral glucose tolerance test; SCFA, short chain fatty acid; SDOH, social determinants of health

* Reflects current readiness for application within a precision nutrition framework in maternity healthcare

** Observed in non-pregnant populations

### Maternal Observational Traits and Social Determinants of Health

#### Age and Parity

Maternal age and parity are important risk stratification factors in pregnancy and may be particularly relevant among women with obesity. Positive interaction effects between advanced maternal age and pre-pregnancy overweight/obesity have been demonstrated for e.g., preeclampsia and GDM risk [18]. Compared with normal-weight women < 35 years, GDM risk was 1.8-fold higher in women < 35 years with overweight/obesity and 2.8-fold higher in those > 35 years with overweight/obesity [19]. Additionally, parity has been linked to insulin resistance and type 2 diabetes, with overweight and obesity aggravating these associations [20, 21]. However, most evidence stems from retrospective or small observational cohorts, and a large individual participant data meta-analysis concluded that neither age nor parity modified the effectiveness of lifestyle interventions in reducing GWG [22]. Within a precision nutrition framework, age and parity are therefore more appropriately considered stratification variables rather than direct targets for dietary tailoring, likely reflecting underlying metabolic and behavioral heterogeneity.

#### Pre-pregnancy Body Mass Index (BMI)

Pre-pregnancy BMI is currently used in clinical guidelines to set GWG targets [23]. However, these recommendations only distinguish between overweight and obesity, without considering obesity severity across established classes, and there is also a lack of energy intake recommendations for these groups [24]. This lack of granularity limits personalization and highlights an opportunity for developing precision nutrition approaches. Obesity class is a strong predictor of pregnancy risks: higher BMI categories are linked to progressively elevated risk for GDM, hypertensive complications, cesarean delivery, LGA infants [25], stillbirths, and congenital anomalies [26]. Furthermore, a systematic review and meta-analysis (54 studies, 30.2 million pregnancies) found that GWG below current recommendations had more favorable obstetrical and neonatal outcomes across all obesity classes [27]. In addition, population-based cohort data indicate that GWG below guidelines or weight loss does not increase risks for adverse maternal and neonatal outcomes in women with obesity class I or II. Moreover, in women with obesity class III, GWG below current recommendations was associated with reduced risk of a composite outcome of adverse maternal and infant events (adjusted RR 0.81, 95% CI 0.71–0.89) [5]. Indeed, revised recommendations that incorporate obesity class [5, 27–29], aligning with precision nutrition principles of stratification and personalization are called for. However, it is important to note that new approaches to diagnosing and defining obesity are emerging. The 2025 *Lancet Diabetes & Endocrinology* Commission [30] argued that BMI frequently leads to misclassification and should not be used as a surrogate marker of an individual’s health. Excess adiposity should instead be confirmed through body fat assessment or complementary anthropometric measures, interpreted together with clinical data and tests indicating tissue or organ alterations, to classify clinical, preclinical or non-clinical obesity [30]. This new definition is important although more research is required to reveal whether adverse pregnancy outcomes differ between women with clinical, pre-clinical and non-clinical obesity. These developments may inform future stratified dietary approaches in gestational obesity, although interventional evidence remains limited.

#### Ethnicity and Social Determinants of Health

Maternal ethnicity is associated with distinct risk profiles for adverse gestational outcomes. For example, population-based data suggests that excessive GWG is a stronger predictor of LGA than pre-pregnancy BMI in White and Asian women, while their effects are similar in Black women [31]. A large meta-analysis further emphasized the use of region-specific BMI classifications in Asian populations to assess excessive GWG according to the National Academy of Medicine’s guidelines as it will affect the prevalence of excessive GWG substantially (51% vs. 37% for regional versus the universal WHO BMI categories) [32]. While these findings support ethnicity-tailored BMI categorization and corresponding adjustments to GWG recommendations, more research is needed to determine how country of residence and migration status may modify these differences.

Social determinants of health (SDOH) are also important to consider as they contribute to disparities in pre-pregnancy obesity [33], modify the risk of adverse pregnancy outcomes [34, 35], and pose challenges for the implementation and effectiveness of lifestyle interventions. Factors such as food insecurity have been linked to higher rates of pre-pregnancy obesity and increased risk of perinatal complications, while food provision assistance during pregnancy has been shown to mitigate these risks [36]. Other SDOH also warrant exploration, for instance migrant women may face language barriers and limited access to traditional foods, potentially hindering adherence to dietary and GWG recommendations [37, 38]. These determinants vary across countries and regions, underscoring the need for context specific approaches. In practice, ethnicity and SDOH primarily support culturally and context-adapted delivery of guideline-based dietary advice, rather than individualized interventions.

### Behavioral Factors

#### Diet

Dietary patterns are key components within the precision nutrition framework, influencing metabolic states and interacting with biological mechanisms [8]. Assessing dietary behavior early in pregnancy can provide a practical starting point for risk stratification and may complement molecular profiling to inform targeted strategies. Behavioral-metabolic obesity phenotypes (i.e., hungry brain, emotional hunger, hungry gut, and slow burn) [39] illustrate the heterogeneity of obesity and reinforce the need for subgroup risk stratification. For example, women with a “hungry brain” phenotype may benefit from appetite regulation strategies, whereas those with a “slow burn” phenotype may respond better to timing-based approaches and macronutrient adjustments. Chrononutrition is also gaining attention, with maternal nighttime eating being associated with adverse pregnancy outcomes [40] and identified as a potential target for improving glycemic control in GDM [41]. However, evidence from pregnancy-specific trials evaluating phenotype-guided dietary strategies is currently limited, and these constructs should be considered hypothesis-generating.

#### Movement Behaviors

Movement behaviors (physical activity, sedentary time, and sleep) are important to consider, with evidence suggesting that specific activity types, patterns, and doses confer distinct metabolic benefits during pregnancy. A meta-analysis in women with overweight and obesity found that moderate‑intensity exercise beyond walking alone reduced gestational hypertension risk by 62% [42]. Similarly, a meta-analysis in women with GDM demonstrated significant improvements in glycemic control, with reduced fasting glucose (−0.47 mmol/L), 2-hour postprandial glucose (−0.62 mmol/L), and HbA1c (−0.39%) with prescribed exercise versus with routine care [43]. Subgroup analyses indicated greater glycemic reductions with shorter-duration and more frequent sessions [43]. Dose-response analyses further indicate that achieving 10 MET‑h/week in early pregnancy reduces GDM risk by 13%, and higher activity volumes (20–50 MET‑h/week) confer further reductions [44]. In addition, other movement‑related behaviors, particularly sleep and sedentary time, are associated with metabolic regulation. In a cohort of pregnant women, sleep duration showed a U‑shaped association with glycemia, and greater sedentary time was associated with higher 1‑hour post‑load glucose (β = 0.132; *P* = 0.005) [45]. While these data support risk stratification based on physical activity patterns, more research is needed to elucidate how they can guide and complement individualized dietary strategies.

### Clinical Biomarkers

#### Metabolic Heterogeneity and Biomarker Stratification

The emerging concept of clinical obesity moves beyond traditional BMI-based definitions and incorporates functional disturbances as part of the illness-defining criteria [30]. This perspective aligns with the growing recognition that metabolic heterogeneity is highly relevant in gestational obesity management [46]. A recent review [47] highlights that maternal obesity is associated with endocrine, metabolic, and inflammatory disturbances, reflected in biomarkers such as leptin, adiponectin, insulin, progesterone, hCG, and CRP. These biomarkers have been proposed as early predictors of e.g., preeclampsia and GDM and may provide a biologically grounded basis for risk stratification. Niclou et al. [46] described an adverse metabolic milieu, marked by elevated glucose, insulin, triglycerides, leptin, and inflammatory markers as a potential basis for stratifying pregnant women with obesity into subgroups. They further suggest that dietary strategies such as time-restricted eating could enhance metabolic flexibility and insulin sensitivity [46], although evidence in pregnancy remains limited [48]. Beyond targeted biomarkers, untargeted multiplatform metabolomics combined with machine learning has also been used in obesity research to identify metabolites predictive of dietary intervention responses. For example, reduced urinary adipic acid and argininic acid levels have been associated with greater responsiveness to a New Nordic Diet in non-pregnant populations [49]. However, these approaches remain largely unexplored in pregnancy, and their translation into actionable dietary strategies for gestational obesity is not yet supported by interventional evidence. The following section provides a more detailed examination of already routinely measured clinical biomarkers.

#### Glucose Parameters

Glucose assessment is particularly important among women with gestational overweight or obesity, given their elevated risk of glucose dysregulation. Therefore, the American Diabetes Association recommends screening for undiagnosed prediabetes or diabetes in women with overweight or obesity who are planning a pregnancy or who are < 15 weeks’ gestation and have one or more additional risk factors (e.g. first-degree relative with diabetes, hypertension, polycystic ovary syndrome [PCOS], physical inactivity), or even considering testing all women [50]. For women who have not been diagnosed earlier, screening for GDM in gestational week 24–28 is recommended. Identifying hyperglycemia early is essential, given that it is associated with maternal and neonatal complications [51–53] with excessive fetal growth being one of the main underlying mechanisms. Medical nutrition therapy is the first-line therapy for GDM [54] and has been shown to improve maternal glycemic control, reduce the risk for medical treatment by 35%, and lower infant birth weight [55]. The need for insulin therapy, which indirectly reflects an insufficient response to nutritional therapy, can be predicted by several factors, including a history of prior GDM, higher BMI, chronic hypertension, or elevated glucose values [56]. However, data on the optimal and potential subgroup-specific dietary approach and obesity management for hyperglycemia during pregnancy are lacking, and several questions such as optimal carbohydrate intake and best modification of dietary interventions remain unanswered.

#### Lipid Profile

Obesity strongly predisposes to dyslipidemia also during pregnancy, which is associated with increased risk of complications, such as preeclampsia, GDM, and pre-term delivery, as well as pancreatitis in the presence of severe hypertriglyceridemia [57, 58]. The 2024 European Society of Cardiology guidelines on dyslipidemia management in pregnancy note that there are no tailored dietary directives for this population. Instead, they recommend a similar diet as for non-pregnant individuals, emphasizing an overall healthy dietary pattern with reduced intake of animal derived foods, processed foods and unhealthy fats (saturated- and trans fatty acids), and increased intake of fruit and vegetables and healthy fats (omega-3 and polyunsaturated fats). The guidelines also state that pregnant women should avoid overly restrictive diets [59].

#### Blood Pressure

Hypertensive disorders of pregnancy (essential hypertension, pregnancy-induced hypertension, and preeclampsia) are strongly associated with adverse maternal and perinatal outcomes, such as preterm birth, fetal growth restriction, and increased maternal morbidity [60]. These conditions are more prevalent among women with obesity, highlighting the need for targeted preventive strategies. Evidence suggests that adherence to the Dietary Approaches to Stop Hypertension (DASH) diet (high intake of fruits, vegetables, wholegrains, low-fat dairy, and low in animal protein, sugar and sodium) in a low-risk pregnant population is associated with lower mid-pregnancy diastolic blood pressure (− 0.45 mmHg per SD increase in DASH score; 95% CI: −0.78 to − 0.12) and improved fetoplacental vascular function [61]. Although modest effects, this indicates that nutritional interventions tailored to blood pressure profiles, such as promoting DASH-like dietary patterns could be relevant to consider.

### Tissue-/organ Specific Level Features

Two examples of organ-specific characteristics relevant for precision nutrition in pregnancy include PCOS and prior bariatric surgery. Women with PCOS, particularly the OB-PCOS subtype characterized by metabolic dysfunction, have an elevated risk of GDM and hypertensive complications [62], and existing evidence suggests that combined diet and physical activity interventions for GDM prevention may be less effective in this group [63]. Whether different PCOS subtypes respond differently to specific dietary strategies during pregnancy remains unclear and warrants further investigation. Women with prior bariatric surgery represent another group requiring tailored nutritional management. Although surgery reduces obesity-related risks, these women remain vulnerable to micronutrient deficiencies (e.g., iron, folic acid, vitamin B12, vitamin D, calcium, thiamine) [64–66], and guidelines recommend enhanced monitoring and supplementation, as well as higher-dose folic acid for women with prior bariatric surgery and BMI > 30 kg/m² during the periconception period and first trimester [67]. Individualized nutritional counselling may help optimize diet quality and support healthy foetal growth in post-bariatric pregnancies [66].

### Molecular-level Data

#### Genetic Predisposition and Nutrigenetics

Genetic factors such as predisposition to central fat distribution and high polygenic risk scores have been associated with increased risks of pre-eclampsia, GDM, and hypertensive disorders [68–70]. Nutrigenetics further highlights how genetic variability can modify dietary responses, with evidence from non-pregnant populations showing that the effects of polyunsaturated fat, reduced saturated fat, and omega‑3 intake on lipid and cardiometabolic profiles differ by genotype [71]. Nutrigenetics also extends to micronutrient responses; for example, individuals with the MTHFR 677TT genotype show blood pressure reductions with riboflavin supplementation [72], although its relevance for hypertensive disorders in pregnancy remains unclear. Large European trials [73, 74] show that gene-based personalized nutrition advice can improve diet quality, but clinical benefits have not been demonstrated, and no pregnancy trials have shown improved outcomes from genetically guided dietary interventions compared with standard guidelines.

#### Gut Microbiota

Women with obesity or GDM often exhibit gut microbiota dysbiosis, characterized by reduced microbial diversity and shifts in short‑chain‑fatty‑acid–producing and glucose‑metabolism–related microbial communities [75–77]. Evidence suggests that probiotic supplementation can improve glucose and insulin levels in women with GDM, whereas effects are absent in those without [78]. Diet quality also shapes microbial composition, with higher‑fiber diets supporting a healthier profile [79]. However, despite these associations, microbiota‑guided dietary strategies are not yet established for pregnancy.

### Stakeholders’ Perspectives - What are Women and Health Care Providers’ Perceptions on Precision Nutrition?

To our knowledge, no studies have explored women’s perceptions of precision nutrition for gestational obesity management, or the intervention features they would appreciate. In fact, current evidence on women’s perspectives on gestational obesity management in maternity healthcare *in general* is limited [80]. Existing studies suggest that women with obesity frequently experience weight stigma when healthcare professionals avoid discussing weight, make assumptions about lifestyle behaviors, or communicate obesity-related risks in an inadequate or overly intense way [81]. Women also report feeling judged because of their weight, receiving inconsistent advice and care, and experiencing poor continuity throughout pregnancy [80]. To address these concerns, staff sensitivity training, patient-centered approaches and educational resources for women have been proposed [81–83]. Digital interventions are also promising, with evidence suggesting that monitoring and receiving feedback on healthy GWG may be beneficial [84]. These gaps highlight the urgent need to explore women’s own preferences and acceptability of precision nutrition approaches for managing gestational obesity. Correspondingly, evidence on healthcare professionals’ practices is limited. Qualitative studies suggest insufficient dietary advice from healthcare in pregnancy [85, 86]. For instance, a Swedish study found that 17% of midwives avoid weight-related discussion to prevent pregnant women from feeling worried or ashamed, a tendency more common among those without training in motivational interviewing [87]. Additional barriers include time-constraints, limited cultural competence, and lack of training in lifestyle counseling and existing guidelines, leading to inconsistent guidance and missed opportunities for early intervention [38, 88]. To summarize, advancing precision nutrition in routine care will require addressing these challenges, improving communication strategies related to obesity *in general* and developing culturally tailored approaches for marginalized populations (e.g. improved cultural knowledge).

### Technological Requirements and Readiness for Precision Nutrition Approaches

Another key aspect to consider is technological requirements and readiness for precision nutrition approaches. Both risk profiling and tailored interventions will likely rely on digital platforms to enable data collection, processing, and resource-effective intervention delivery. Digital solutions for precision nutrition in non-pregnant populations are advancing rapidly [89]. For interventions, we can build on over 15 years of research using digital technologies (e.g., smartphone apps, wearable technologies) to support lifestyle behavior change [90]. Such applications have also demonstrated effectiveness in GWG management among women with obesity [91]. Furthermore, Artificial Intelligence (AI) offers significant potential for risk modelling, stratification, and enhancing intervention content, flexibility and delivery. For instance, AI tools can generate tailored content to address diverse needs (e.g., languages, literacy levels) and provide tailored dietary and physical activity advice based on real-time data (e.g., blood glucose values, uploaded meal-pictures, acceleration data or steps). Regarding cultural adaptations of digital interventions, tailoring content to linguistic and cultural needs appears promising for increasing reach and engagement among diverse populations [92]. Moreover, digital health solutions have potential in addressing SDOH by increasing access to e.g. dietary counselling and monitoring in pregnancy, potentially replacing or supplementing in-person visits and reducing barriers related to time, distance and resource constraints. In summary, *technological readiness* for precision nutrition is high, enabling the development and delivery of interventions based on large, granular datasets - once there is greater clarity on which variables to include.

### Realization of Precision Nutrition into Routine Care

Based on our summary above, it is reasonable to conclude that realizing the outlined precision nutrition framework (Fig. 1A) at its full potential in routine care remains a distant goal. Several factors contribute to this gap, including limited evidence from randomized controlled trials evaluating specific precision nutrition approaches and low feasibility in maternity care where many relevant variables are not routinely assessed (Table 1) and may remain difficult to measure due to time and financial constraints. Moreover, although technological readiness (e.g., digital health platforms that enable data collection on lifestyle behaviors and physiological variables) is high, users’ needs and perceptions are still largely unexplored. Consequently, existing evidence does not support full-scale implementation of biologically tailored dietary prescriptions, which remain largely conceptual and primarily hypothesis-generating. Potential future steps are instead illustrated in Fig. 1B and may encompass improved risk stratification based on clinical assessment (level I), followed by more individualized lifestyle counselling and GWG targets based on extended initial screening, which can include existing dietary and physical activity behaviors as well as obesity phenotypes (level II). A comprehensive multi-level approach incorporating omics data (level III) may be viewed as a future vision for gestational obesity management, analogous to general obesity treatment.

### Need for More Research

To move forward with more precision-based nutrition approaches that can be implemented in routine care for gestational obesity management (Fig. 1B), several key research gaps need to be addressed (Table 2). Future research should prioritize identifying and validating dietary patterns, such as time-restricted eating or tailored food and macronutrient compositions, that can optimize outcomes for subgroups defined by obesity classification (clinical/non-clinical obesity [30]), metabolic milieu [46] or behavioral-metabolic phenotype (e.g., hungry brain, emotional hunger, hungry gut [39]). One important effort which will provide valuable insights is the ongoing NIH precision for nutrition health trial, which includes a representative sample of the U.S. population, also including pregnant women (https://commonfund.nih.gov/nutritionforprecisionhealth). There is also an urgent need for well-designed randomized controlled trials to evaluate the efficacy of more precision-based approaches on weight management and adverse maternal and neonatal outcomes. This could include tailored interventions targeting certain subgroups or individuals by collecting and utilizing multi-level data including real-time measures of dietary and physical activity behaviors combined with wearables (e.g., accelerometers, continuous glucose monitoring) as well as more granular measures such as multi-omics. To facilitate translation into routine care, digital health interventions are likely to be prioritized, and designs like Sequential, Multiple Assignment Randomized Trials, and micro-randomized trials [93, 94] may be considered to improve personalization and outcomes. In parallel, research should address end-users’ (women, healthcare professionals and healthcare services) needs and perceptions, and examine implementation aspects (e.g., acceptability, feasibility, engagement and cost-effectiveness), where hybrid trial designs combining effectiveness and implementation aspects may be relevant [92]. For successful implementation, an early implementation plan, a clear business model, continuous stakeholder engagement, future-proof digital technology and culturally and linguistically inclusivity are key recommendations [95].

**Table 2:** Summary of research needed to advance precision nutrition in gestational obesity management

Research area	Key research questions to address	Proposed study design
Pathophysiology and precision factors	What non-modifiable and modifiable factors should be assessed and used as indicators and targets for tailored interventions? Confirming and expanding existing knowledge.	Large-scale, prospective, and representative cohort studies collecting multi-dimensional data on factors like diet, genetics, microbiota, clinical biomarkers, and SDOH* from different countries. Well-controlled meal studies
Women’s perspectives and needs	How do women perceive precision nutrition tools for gestational obesity management? What features would they like? What data would they like to measure and share? What technology would they prefer?	Qualitative studies
Healthcare professionals’ perspectives and needs	How would healthcare professionals perceive the use of precision nutrition tools for gestational obesity management in their daily work? What variables would they like to screen for and monitor and how?	Qualitative studies
Healthcare services’ perspectives and needs	What is the technological readiness for precision nutrition tools? What technologies can be used? What reimbursement and business models can be applied?	Qualitative studies
Effectiveness – and cost-effectiveness of developed precision tools on clinical outcomes and engagement	What is the (cost)- effectiveness based on GWG, and maternal and neonatal outcomes (e.g., incidence of GDM, pregnancy-induced hypertension, pre-eclampsia, mental health issues, LGA infants, caesarean sections, neonatal care and breastfeeding initiation)?	Randomized controlled trials (potentially hybrid designs)
Implementation aspects	How acceptable, appropriate, and feasible are interventions for healthcare professionals, and what is their reach? Additionally, what are women’s levels of satisfaction and patterns of use?	Implementation outcomes (e.g., as part of hybrid designs) using qualitative and quantitative data
Societal relevance	How does weight stigmatization and movements around body positivity impact women’s engagement in gestational obesity management programs? How can this be handled?	Qualitative studies

*GWG* gestational weight gain, *GDM* gestational diabetes, *LGA* large-for-gestational age, *SDOH* social determinants of health

*For more details on data levels, see Table 1

## Conclusion

While this review highlights that significant work remains to fully realize the potential of comprehensive multi-level precision nutrition for gestational obesity management, we strongly encourage researchers and clinicians to lead efforts in advancing this field into more precision-based interventions and care-models moving beyond current one-size-fits all. Developing such models has the potential to enhance maternity care and improve health outcomes for women and children.

## Key References

- da Silva BR, Brennan L, Horst MA, Wishart DS, Prado CM. Advancing precision nutrition: bridging mechanistic insight and clinical implementation. Nat Rev Endocrinol 2025:21:515–17. Doi:10.1038/s41574-025-01141-9.
  - ***○ ***Structured framework to guide precision nutrition studies.
- Johansson K, Bodnar LM, Stephansson O, Abrams B, Hutcheon JA. Safety of low weight gain or weight loss in pregnancies with class 1, 2, and 3 obesity: a population-based cohort study. Lancet 2024:403:1472–81. Doi:10.1016/S0140-6736(24)00255-1.
  - ***○ ***Weight loss in women with obesity not associated with increased risk for adverse maternal or neonatal outcomes. Revised GWG guidelines are warranted taking obesity class into consideration.
- Niclou AM, Cabre HE, Flanagan EW, Redman LM. Precision Interventions Targeting the Maternal Metabolic Milieu for Healthy Pregnancies in Obesity. Curr Diab Rep 2024:24:227 − 35. Doi:10.1007/s11892-024-01550-6.
  - ***○ ***Focus on maternal metabolic milieu rather than GWG as target in women with obesity.
- Dever M, Skouteris H, Incollingo Rodriguez AC, Hailu H, Galvin E, Hill B. Weight stigma in the preconception, pregnancy, and postpartum periods: A systematic review of women’s perspectives. Obes Rev 2025:26:e13891. Doi:10.1111/obr.13891.
  - ***○ ***More research on women’s perspectives on weight management in maternity care is required.
- Löf M, Maddison R. Implementing digital health to support self-care of chronic diseases. Nat Med 2025:31:2093–2094. Doi:10.1038/s41591-025-03729-0.
  - ***○ ***Six recommendations to translate digital health tools from research to routine care.

## Data Availability

No datasets were generated or analysed during the current study.
